# *M. tuberculosis* Hypothetical Proteins and Proteins of Unknown Function: Hope for Exploring Novel Resistance Mechanisms as well as Future Target of Drug Resistance

**DOI:** 10.3389/fmicb.2017.00465

**Published:** 2017-03-21

**Authors:** Divakar Sharma, Deepa Bisht

**Affiliations:** Department of Biochemistry, National JALMA Institute for Leprosy and Other Mycobacterial Diseases,Agra, India

**Keywords:** *M. tuberculosis*, proteomics, bioinformatics, hypothetical proteins, mechanism of drug resistance

## Abstract

Drug resistance in tuberculosis predominantly, mono-resistance, multi drug resistance, extensively drug resistance and totally drug resistance have emerged as a major problem in the chemotherapy of tuberculosis. Failures of first and second line anti-tuberculosis drugs treatment leads to emergence of resistant *Mycobacterium tuberculosis.* Few genes are reported as the principal targets of the resistance and apart from the primary targets many explanations have been proposed for drug resistance but still some resistance mechanisms are unknown. As proteins involved in most of the biological processes, these are potentially explored the unknown mechanism of drug resistance and attractive targets for diagnostics/future therapeutics against drug resistance. In last decade a panel of studies on expression proteomics of drug resistant *M. tuberculosis* isolates reported the differential expression of uncharacterized proteins and suggested these might be involved in resistance. Here we emphasize that detailed bioinformatics analysis (like molecular docking, pupylation, and proteins-proteins interaction) of these uncharacterized and hypothetical proteins might predict their interactive partners (other proteins) which are involved in various pathways of *M. tuberculosis* system biology and might give a clue for novel mechanism of drug resistance or future drug targets. In future these uncharacterized targets might be open the new resistance mechanism and used as potential drug targets against drug resistant tuberculosis.

## Introduction

### Current Scenario

Tuberculosis (TB) still remains one of the deadliest infectious diseases worldwide which is caused by *Mycobacterium tuberculosis.* WHO reported 10.4 million people became ill and that 1.8 million died from TB (WHO, 2016). For controlling this situation the available current tools are vaccine diagnostics and drugs. Over the past 50 years, the *Mycobacterium bovis* bacille Calmette–Guérin (BCG) vaccine against TB has maintained its position as the world’s most widely used vaccine, despite showing highly variable efficacy (0–80%) in different trials (Andersen and Doherty, 2005).

Sputum smear microscopy is the most common TB diagnostic method worldwide. However, culture remains the gold standard and the use of rapid molecular testing like line probe assay (LPA) is increasing for detection of drug resistant *M. tuberculosis* strains. Recently in India, Revised National TB Control Program (RNTCP) has approved a study for the Validation of second line LPA for detecting resistance to fluoroquinolones, aminoglycosides (kanamycin, amikacin), and cyclic peptides (capreomycin). First and second line drugs are the effective and necessary component of short course chemotherapy. DOTS and DOTS plus program have reduced the incidence of TB caused by susceptible strain but emergence of multidrug-resistant tuberculosis (MDR-TB), extensively drug resistant tuberculosis (XDR-TB), and totally drug resistant tuberculosis (TDR-TB) have worsened the situation and became a major threat to public health. Current tools (vaccines, diagnostics, and therapeutics) are unable to offer the complete protection against these deadly drug resistant situations.

### Mystery behind the Drug Resistance

**FIGURE 1:**
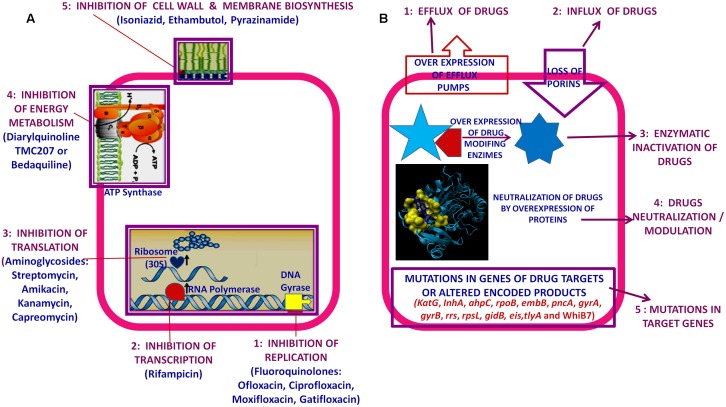
**Schematic diagram showed: (A)** Potential mechanism (s) of action of first and second line of anti-TB drugs. **(B)** Potential mechanism (s) of drug resistance in *Mycobacterium tuberculosis.*

Usually interrupted anti-TB drugs treatment (first and second line anti-mycobacterial drugs) leads to emergence of drug resistant *M. tuberculosis* strains. Probably these resistant *M. tuberculosis* strains can resist antibiotic actions by the series of mechanisms such as: mutations in target genes (Beauclerk and Cundliffe, 1987), enzymatic inactivation of antibiotic molecules (Welch et al., 2005), over expression of novel efflux pumps and porin alterations in the cell wall (Magnet et al., 2001; Nikaido, 2003), trapping of drugs and the over expression of proteins involved in neutralizing the effect of drugs (Magnet et al., 2003; Kumar et al., 2013; Lata et al., 2015a; Sharma et al., 2015a, 2016b). Genes involved in these drug resistance mechanisms are tabulated in **Table 1** (adopted from Zhang and Yew, 2009 and updated). **Figure 1** is the schematic diagram which showed the potential mechanism (s) of action of first line (Rifampicin-inhibition of RNA synthesis, Isoniazid-inhibition of mycolic acid biosynthesis, Ethambutol-inhibition of arabinogalactan synthesis, Pyrazinamide-depletion of membrane energy, and Streptomycin-inhibition of protein synthesis) and second line of anti-TB drugs (Amikacin, Kanamycin, Capreomycin-inhibition of protein synthesis and Quinolones-inhibition of DNA gyrase) and the potential mechanism (s) of drug resistance, respectively. These mechanisms of action of drugs were also tabulated in **Table 1**. Usually 36–95% resistances in *M. tuberculosis* were contributed by mutations in the target genes, however, remaining 5–64% does not have these mutations and signifying the contribution of some other resistance mechanism (s). Research through expression proteomics (2D gel electrophoresis) and bioinformatic tools (like molecular docking, pupylation, and proteins-proteins interaction) explored the other novel mechanisms of drug resistance were accumulated in last decade and still underway. Patch dock and fire dock (molecular docking) predicted, drug binds to the conserved domain of the hypothetical proteins and suggested that overexpression of these proteins of undefined role might be neutralize/modulate the effects of drugs (Sharma et al., 2015a, 2016b). Pupylation is a post translational modification through which small disordered protein Pup is conjugated to lysine residues of proteins marking them for proteasomal degradation. GPS-PUP (pupylation) predicted, that neutralized/modulated adduct (drug-protein complex) might be degraded by proteasome machinery complex {turnover of the proteins} (Sharma et al., 2015a, 2016b). As modification with pup is reversible, pupylation is also likely to have a regulatory role. Pup-proteasome system controlled by pupylation contributes to the virulence/survival strategy of *M. tuberculosis* in the host and makes the bacteria more resistant to various stresses. STRING-10 (Proteins-proteins interaction) predicted the potential interactive partners and suggested the metabolic pathways involved in resistance (Sharma et al., 2016a,b). Rv0148 (hypothetical protein/putative short-chain type dehydrogenase/reductase) was overexpressed in aminoglycosides resistant *M. tuberculosis* and had conserved SDR domain. We found that aminoglycosides binds to SDR domains and might be neutralized the drug effect (Sharma et al., 2015a). Further we characterized it and found that overexpression of Rv0148 involved in shift in MIC of aminoglycosides in recombinant *E. coli* (Sharma et al., 2015b). These findings might be used in the development of newer therapeutic agents or molecular markers which can directly be targeted to a gene/protein responsible for resistance. In this work we discuss the probable involvement of uncharacterized/hypothetical proteins (differential expression in drug resistance studies were previously reported) in drug resistance via bioinformatics analysis (like molecular docking, pupylation, and proteins-proteins interaction) and suggested that these might be explore novel mechanism of drug resistance or used as potential future drug target/diagnostics against drug resistance.

**Table 1:** Mechanisms of genes involved in drug resistance in *Mycobacterium tuberculosis.*

Drug (Year of discovery)	MIC (μg/ml)	Gene (s) involved in resistance	Gene-product	Mechanism of action	Mutation frequency %
Isoniazid, 1952	0.02–0.2	*kat*G*inh*A	Catalase-peroxidase Enoyl ACP reductase	Inhibition of mycolic acid biosynthesis and other multiple effects	50–95 8–43
Rifampicin, 1966	0.05–1	*rpo*B	β-subunit of RNA polymerase	Inhibition of RNA synthesis	95
Pyrazinamide, 1952	16–50 (pH 5.5)	*pnc*A	Nicotinamidase/pyrazinamidase	Depletion of membrane energy	72–97
Ethambutol, 1961	1–5	*emb*B	Arabinosyl transferase	Inhibition of arabinogalactan synthesis	47–65
Streptomycin, 1944	2–8	*rps*L*rrs gid*B	S12 ribosomal protein 16S rRNA rRNA methyltransferase	Inhibition of protein synthesis	52–59 8–21 ?
Amikacin Kanamycin, 1957; Capreomycin, 1960	2–4	*Rrs tly*A	16S rRNA 2′-*O*-methyltransferases	Inhibition of protein synthesis	76
Quinolones, 1963	0.5–2.5	*gyr*A*gyr*B	DNA gyrase subunit A DNA gyrase subunit B	Inhibition of DNA gyrase	75–94
Ethionamide, 1956	2.5–10	*eth*A	Flavin monooxygenase	Inhibition of mycolic acid synthesis	37
PAS, 1946	1–8	*thy*A	Thymidylate synthase	Inhibition of folic acid synthesis and iron metabolism	36
Bedaquiline, 2012	0.125–0.50	*atp*E	ATP synthase	Block the proton pump for ATP synthesis	?

*MIC, minimum inhibitory concentration; ACP, acyl carrier protein; PAS, Para-aminosalicylic acid.*

### Expression Proteomics and Bioinformatics Approaches: A Way for Exploring the Mystery of Drug Resistance

Advancement in proteomics has explored the mystery behind any complex phenotypes like drug resistance. As proteins manifest most of the biological processes, these are attractive targets for exploring new mechanisms of the drug resistance. Expression proteomics (2-DE coupled with MALDI-TOF-MS) and bioinformatic tools {patch dock and fire dock for molecular docking, GPS-PUP for pupylation, and STRING-10 for protein-protein interaction} have now emerged as major analytical tools for identification and characterization of expression proteome (proteins and its species) (Sharma et al., 2015a; Sharma and Bisht, 2016). In the last decade (2006–2016) few reports on expression proteome of drugs resistant *M. tuberculosis* existed (Jiang et al., 2007; Sharma et al., 2010, 2014, 2015b, 2016b; Kumar et al., 2013; Lata et al., 2015a,b; Singh et al., 2015; Sharma and Bisht, 2017) and suggested that differential expression of functionally known and unknown proteins and their protein-protein interaction might be involved in resistance and could be explore the novel mechanism of resistance.

## Hypothetical Proteins and Proteins of Unknown Function: Potential Targets of Drug Resistance or Novel Resistance Mechanisms

Since last decade, few proteomics and bioinformatics studies of drug resistant *M. tuberculosis* have been accumulated and reported the differential expression of a panel of uncharacterized (proteins of unknown function) and hypothetical proteins. Through *in silico*/bioinformatic (Interproscan and molecular docking) analysis they showed that drugs binds to the conserved domains of hypothetical proteins/uncharacterized proteins and suggested that the over expression of a panel of uncharacterized and hypothetical proteins might neutralize/compensate the effect of drugs. Sharma et al. (2015b, 2016a) reported that inducible over expression of cloned known and unknown proteins (Rv0148 and Rv3841) in *E. coli* makes it two- to threefolds more resistant under drug pressure. Here we emphasize that detailed bioinformatics analysis (like protein-protein interaction) of these uncharacterized and hypothetical proteins might predict their interactive partners (other proteins) which are involved in various pathways of *M. tuberculosis* system biology (exploring/deciphering the *M. tuberculosis* network biology through *in silico*/holistic approaches) and might give a clue for novel mechanism of drug resistance or future target. Research in this direction could prevent the emergence of MDR-TB, XDR-TB, and TDR-TB situation and also these targets may be used to discover the new drug entities as potential drug candidates against drug resistant tuberculosis.

## Conclusion and Future Perspective

Based on the evidence discussed above related to expression proteomics and bioinformatics studies of drug resistant *M. tuberculosis* have been reported the differential expression of uncharacterized and hypothetical proteins (Jiang et al., 2007; Sharma et al., 2010, 2014, 2015b, 2016b; Kumar et al., 2013; Lata et al., 2015a; Singh et al., 2015; Sharma and Bisht, 2017). Molecular docking analysis of these uncharacterized and hypothetical proteins suggested that their over expression might neutralize/compensate the effect of drugs (Sharma et al., 2010, 2015a, 2016b; Kumar et al., 2013; Lata et al., 2015a; Sharma and Bisht, 2017) which further proved by other reports and showed inducible over expression of cloned known and unknown proteins in *E. coli* which makes it few folds more resistant under drug pressure (Sharma et al., 2015b). Here we emphasized the detailed *in silico* analysis through patch dock, fire dock (molecular docking), GPS-PUP (pupylation), and STRING-10 (predicts the proteins-proteins interactions/interactome) of these uncharacterized and hypothetical proteins which might be involved in various pathways of *M. tuberculosis* system biology (Lata et al., 2015a; Sharma et al., 2015a, 2016b; Sharma and Bisht, 2017). Future research in this direction could be uncovering the novel mechanism of drug resistance or future target. Which ultimately prevent the emergence of deadly resistant strains of *M. tuberculosis* and could leads to discovery of the new drug entities against deadly drug resistant tuberculosis.

## Author Contributions

DS design the concept and wrote the manuscript. DS and DB finalized the manuscript.

## Conflict of Interest Statement

The authors declare that the research was conducted in the absence of any commercial or financial relationships that could be construed as a potential conflict of interest.
